# From imaging techniques to laboratory tests: a multimodal stratification for varicocele-associated decreased semen quality

**DOI:** 10.1186/s12610-025-00268-z

**Published:** 2025-06-02

**Authors:** Anmin Wang, Hongyuan Chang, Dicheng Luo, Hao Wang, Hui Lv, Wenxiao Yu, Fu Wang

**Affiliations:** Department of Andrology, Xiyuan Hospital of China Academy of Chinese Medical Sciences, Beijing, 100091 China

**Keywords:** Varicocele, Semen quality, Imaging techniques, Color Doppler ultrasound, Ultrasound elastography, Assessment tools, Varicocèle, Qualité du Sperme, Techniques d’Imagerie, Echographie Doppler couleur, Élastographie par Ultrasons, Outils d’Evaluation

## Abstract

**Background:**

Varicocele adversely affects male fertility by impairing semen quality, yet there remains no unified approach that combines advanced imaging and laboratory markers for clinical stratification. To develop a uniform clinical stratification system, we conducted a review of the literature across major databases.

**Results:**

Shear wave elastography thresholds above 5.235 kPa and spermatic vein diameters exceeding 2.5 mm on color Doppler ultrasound demonstrated strong, independent correlations with decreased sperm concentration, motility, and morphology. Among laboratory tests, elevated follicle-stimulating hormone, low inhibin B, and others were consistently linked to poorer semen quality. Biomarkers such as cysteine-rich secretory protein 3 showed potential.

**Conclusions:**

We propose a multimodal stratification framework integrating evidence‑based cutoffs for shear wave elastography and color Doppler ultrasound with key laboratory indices. Adoption of standardized protocols and consensus guidelines will facilitate personalized diagnosis, improve prognostic accuracy, and guide management of varicocele‑decreased semen quality.

## Résumé

**Contexte:**

La varicocèle affecte négativement la fertilité masculine en altérant la qualité du sperme, mais il n’existe toujours pas d’approche unifiée combinant une imagerie avancée et des marqueurs de laboratoire pour la stratification clinique. Afin de développer un système de stratification clinique uniforme, nous avons effectué une revue de la littérature dans les principales bases de données.

**Résultats:**

Les seuils d’élastographie par ondes de cisaillement supérieurs à 5,235 kPa et les diamètres des veines spermatiques supérieurs à 2,5 mm à l’échographie Doppler Couleur ont montré des corrélations fortes et indépendantes avec une diminution de la concentration, de la motilité et de la morphologie des spermatozoïdes. Parmi les tests de laboratoire, un taux élevé d’hormone folliculo-stimulante, une faible inhibine B étaient systématiquement liés à une moins bonne qualité du sperme. Des biomarqueurs, tel que la protéine sécrétoire 3 riche en cystéine, ont montré un potentiel diagnostique.

**Conclusions:**

Nous proposons un cadre de stratification multimodale intégrant des seuils fondés sur des preuves pour l’élastographie à ondes de cisaillement et l’échographie Doppler couleur avec des indices de laboratoire clés. L’adoption de protocoles standardisés et de directives consensuelles facilitera un diagnostic personnalisé, améliorera la précision du pronostic et guidera la prise en charge de la qualité du sperme diminuée par la varicocèle.

## Introduction

Varicocele (VC) is defined by the presence of dilated veins in the pampiniform plexus with venous reflux [1]. The pathogenesis of VC primarily involves dysfunction of venous valves and return, but the etiology remains incompletely clarification. Contributory factors include age, body mass index, and hereditary susceptibility, among others [2–4]. Epidemiologic investigations demonstrated that it affects approximately 15% to 20% of the general male population, with prevalence rates reaching 30% to 40% in primary male infertility and 69% to 81% in secondary male infertility [5, 6]. Clinical manifestations ranged from asymptomatic to testicular pain, scrotal swelling, and sexual dysfunction, with a substantial proportion of patients exhibiting impaired semen parameters [7]. Venous stasis in VC elevates scrotal temperature and disrupts testicular blood flow, thereby impairing spermatogenesis [8]. Consequently, many asymptomatic patients remain unaware of VC until they undergo semen quality evaluation. Management strategies encompass conservative measures such as vasoactive pharmacotherapy (e.g. Aescuven forte and Diosmetin) or surgical intervention [9].

Semen quality, a fundamental determinant of male fertility (MI), encompasses sperm count, concentration, motility, and morphology. Elucidating the relationship between VC and these semen parameters is essential to understanding its impact on reproductive potential [10]. Nevertheless, the causal link between VC and MI remains controversial, i.e., VC doesn’t invariably lead to infertility. Some patients continue to exhibit abnormal semen parameters even after treatment, while others maintain normal fertility without intervention. Also, the lack of standardized diagnostic criteria for evaluating the impact of VC on semen parameters has led to methodological inconsistencies among studies and variable therapeutic outcomes.

This review aims to classify, compare, and critically evaluate existing diagnostic methods used to assess the relationship between VC and semen parameters. By synthesizing the available evidence, we strive to identify the most reliable approaches and develop comprehensive proposals to predict changes in VC-associated semen quality and early intervention. Additionally, we will evaluate the clinical utility of these tests across various healthcare settings. The goal is to furnish clinicians with evidence-based guidance for more precisely determining the significance of VC as it relates to male fertility.

## Methods

A comprehensive literature search was conducted through January 2025, encompassing four English-language databases (PubMed, Embase, Web of Science, and Cochrane Library) and three Chinese-language databases (China National Knowledge Infrastructure, China Science and Technology Journal Database, and Wanfang Data). The search strategy incorporated reference list examination of identified studies using relevant keywords. The search terminology consisted of three primary categories: (1) varicocele-related terms ("varicocele,""varicoceles,""primary varicocele,""varicocele surgery,""varicocelectomy,""VC"); (2) semen quality indicators ("semen quality,""sperm motility,""sperm morphology,""sperm parameters,""semen analysis,""male infertility,""reproductive health,""male reproductive disorders"); (3) assessment-related terms ("evaluation,""assessment,""value,""correlation,""relevance"). These categories were combined using Boolean operators AND to optimize search specificity.

**Fig. 1:**
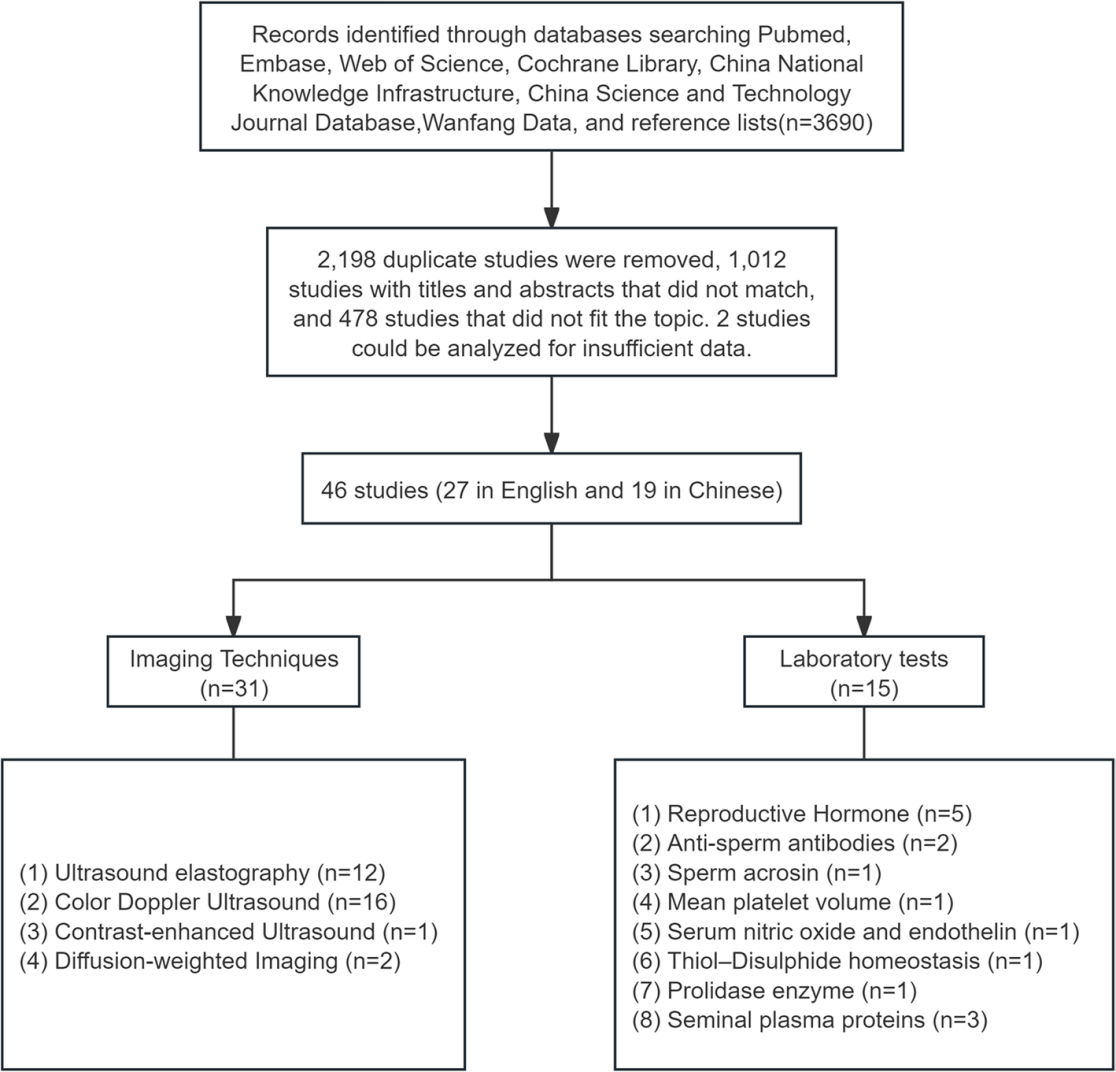
Flow chart of the study search

The initial database search yielded 3,690 documents, among these, we exclude Posters in Congress, Theses, Comments and other documents which have not been submitted to Peer Review Process. Following the removal of 2,198 duplicates and preliminary screening of titles and abstracts, 1,012 studies were excluded. Subsequent full-text review led to the elimination of 478 studies that failed to meet inclusion criteria, primarily due to methodological inconsistencies or inappropriate comparisons. Two additional studies were excluded due to insufficient analyzable data, despite attempts to contact the original authors. The final analysis included 46 studies (27 in English and 19 in Chinese), as illustrated in Fig. 1.

The included studies were stratified into two principal categories based on evaluation methodologies: imaging studies (*n* = 31) and laboratory tests (*n* = 15). Data extraction focused on patient demographics, diagnostic approaches, evaluation methods, and semen parameters. The cumulative study population comprised 3,616 patients with VC, diagnosed by physical examination (PE) and/or ultrasound (US). All participants exhibited varying degrees of decreased semen quality. Semen parameters were standardized to include volume, sperm concentration, count, motility, and morphology.

### Diagnosis of varicocele

VC diagnosis necessitates a comprehensive approach that integrates patient history with PE and, where appropriate, US evaluation in both adolescent and adult populations. Although advanced modalities such as magnetic resonance imaging (MRI) and venography are available, they generally function as adjuncts rather than primary tools to PE and US.

#### Physical examination

PE remains the cornerstone of VC diagnosis and should be conducted under appropriate environmental conditions, including controlled room temperature and patient privacy [11]. The examination protocol encompasses assessment in both supine and standing positions, beginning with visual inspection, followed by palpation and evaluation during Valsalva maneuver. The grading system comprises:

- Grade I: Palpable VC only during Valsalva maneuver
- Grade II: Palpable VC at rest without visual evidence in standing position
- Grade III: Visibly apparent VC in standing position with clear palpable findings [12].

However, PE demonstrates exhibits notable limitations, as demonstrated in a study of 141 men with infertility [13]. The investigation reported detection rates of only 30–40% for PE versus 70% for left-sided venography, along with a 23% false-positive rate. More recent studies indicate that PE sensitivity and specificity are 71% and 69%, respectively, with diagnostic accuracy further reduced by factors such as prior scrotal surgery, edema, or obesity [14]. Although PE remains the primary diagnostic modality, supplemental imaging is advised for cases with equivocal findings or specific symptomatology.

#### Ultrasound evaluation

US is the preferred imaging modality for VC assessment, although its grading systems show only limited correlation with clinical severity. The lack of standardized protocols and diagnostic criteria has led to considerable variability among US grading schemes. A comprehensive review by the European Society of Urogenital Radiology’s Scrotal and Penile Imaging Working Group identified at least ten distinct US grading criteria as of 2016 [15]. The European Association of Urology (EAU) guidelines recommend a four-tier classification system, adding a subclinical VC category to the traditional PE-based grades. Subclinical VC is defined as not palpable or visible at rest or during Valsalva maneuver but detectable on Doppler US [16]. According to the EAU, a venous diameter exceeding 3 mm in the upright position during Valsalva maneuver and a reflux duration over 2 s indicate clinically significant VC [14, 17]. By contrast, the American Urological Association (AUA) and the American Society for Reproductive Medicine (ASRM) practice committees endorse diagnosis based solely on PE [18].

Among the reviewed studies, only one conducted before the widespread availability of US study relied exclusively on PE for VC diagnosis. Although such historical investigations retain some clinical relevance, their methodological constraints may limit their applicability in modern practice [19]. To align with current clinical standards and research methodologies, this review prioritized studies using contemporary diagnostic modalities. Current guidelines differ on whether imaging should be reserved for cases in which PE fails to detect a VC. However, in regions with limited access to trained urologists or where routine specialist referral incurs high costs, reliance on imaging alone can simplify and broaden diagnostic availability. Imaging techniques, particularly CDUS and USE, offer objective, reproducible assessments of venous dilation, reflux patterns, and testicular tissue characteristics. Incorporating such imaging-based algorithms into standard workflows may improve detection rates among men who might otherwise forgo thorough PE, reduce diagnostic disparities, and enable timely management in resource-limited settings.

### Assessment tools between varicocele and semen quality

#### Imaging techniques

Although well established, US imaging technology continues to advance, enhancing its diagnostic potential [20]. Current evidence indicates that US effectively assesses venous blood flow patterns, echogenicity, testicular dimensions, and vascular dilatation in patients with VC. This comprehensive evaluation permits precise characterization of VC severity and subtype, helps distinguish affected from unaffected individuals, and yields critical information for treatment planning [21].

In assessing VC, US focuses on three key parameters: the maximum internal diameter of dilated veins, retrograde venous flow during Valsalva maneuver, and venous tortuosity. Of these, internal vein diameter has emerged as the most reliable diagnostic marker, and the first two parameters are widely accepted as essential criteria [22, 23]. Studies have demonstrated significant correlations between US findings and semen parameters in patients with VC, underscoring US’s value in the evaluation of MI; however, standardized methodologies remain needed [24–26]. Growing recognition of VC's detrimental effects on MI has spurred the development of advanced US modalities, such as CDUS and USE, to more accurately assess its impact on spermatogenesis [27].

#### Ultrasound elastography

USE, first introduced in the 1990 s, represents a major advance in noninvasive tissue stiffness measurement [28]. This modality provides quantitative elasticity metrics, reducing operator-dependent variability and offering objective diagnostic and treatment–response data [29]. The World Federation of Ultrasound Medicine and Biology classifies elastography into three primary types: strain elastography, transient elastography, and acoustic radiation force impulse (ARFI), with ARFI further subdivided into point shear wave, 2D shear wave, and 3D shear wave elastography (SWE) [30]. Although extensively applied in thyroid, prostate, and liver pathology, USE has shown promise in evaluating spermatogenic function in patients with VC [31–33].

Our review of USE studies reveals a significant negative correlation between testicular tissue stiffness and semen quality parameters (Table 1). Fu et al*.* assessed postoperative semen quality improvements in patients with preoperative sperm counts below 5 million/mL using receiver operating characteristic (ROC) analysis [34]. They reported areas under the curve of 0.801 for right testicular volume and 0.775 for bilateral maximum elastic modulus, with respective sensitivities and specificities of 94.6% and 59.8% [95% CI: 0.772–0.867]. Combining these measures enhanced predictive accuracy for sperm concentration. Similarly, Fuschi et al*.* found a significant negative correlation (*r* = −0.422) between reduction in mean left testicular elastic modulus reduction (from 2.56 to 1.79 kPa) and sperm count increase (from 24.17 to 38.06 million/ml) after surgery [35]. Some authors have proposed threshold modulus values exceeding 5.235 kPa for predicting poorer semen parameters, although Liu et al*.* observed normal sperm parameters even at higher elastic modulus values (5.31 ± 0.18 kPa and 6.67 ± 0.12 kPa) in patients with second- and third-degree VC, highlighting the need for further large-scale validation [36, 37]. Other elastographic approaches have yielded similar findings: Lu used virtual touch tissue quantification to measure transverse shear wave velocity (SWV), and Huang demonstrated negative correlations between SWV and both sperm concentration and motility, with semen quality deteriorating when SWV exceeded 0.43 ± 0.09 m/s [38, 39]. He et al*.* reported abnormal sperm density and concentration at mean strain values above 1.67 ± 0.40 and 1.40 ± 0.31, respectively [40], whereas Su et al*.* found only a negative correlation between strain values and VC grade without significant sperm parameter changes [41]. Xie et al*.* noted decreasing sperm concentration with lower left-testis strain ratios correlating with VC severity [42], and Abdelwahab Khaled et al*.* documented a significant negative correlation between the SWE stiffness index and sperm count and total motility, though not morphology [43].

**Table 1:** Ultrasound elastography

Ref	Year	Patients	Mean age (year)	Diagnostic	Evaluation	Semen indicators	Correlation coefficient	
							Sperm concentration	Sperm motility
[34]	2024	568	32.00	PE and Grayscale and CDUS	SWE	Sperm concentration	r _Emax_ = −0.392 r _Emax-L_ = −0.349 r _Emax-R_ = −0.380 r _Emean_ = −0.120 r _Emean-L_ = − 0.086 r _Emean-R_ = − 0.107 r _Emin_ = 0.183 r _Emin-L_ = 0.184 r _Emin-R_ = 0.150	Undescribed
[36]	2024	40	31.34 ± 3.56	CDUS	SWE	Sperm concentration, and sperm motility	r _E_ = −0.391	r _E_ = −0.412
[37]	2022	67	30.00	PE and CDUS	SWE	Sperm concentration, and sperm motility	r _LTSWE_ = −0.262	r _LTSWE_ = −0.555
[35]	2021	82	27.44 ± 6.10	CDUS	SWE	Semen volume, sperm count, total and progressive motility, and sperm morphology	r _LTSWE_ = −0.529	r _LTSWE_ = −0.422
[44]	2021	196	Group 1: 33.17 ± 7.14 Group 2: 31.21 ± 6.97 Group 3: 33.25 ± 7.17	CDUS	USE	Sperm concentration, and sperm motility	r _RI_ = 0.226 r _PI_ = 0.068 r _EDV_ = −0.074 r _PSV_ = −0.077 r _MEAN_ = −0.612	r _RI_ = −0.314 r _PI_ = −0.055 r _EDV_ = 0.292 r _PSV_ = 0.271 r _MEAN_ = −0.678
[38]	2020	120	28.40 ± 4.10	PE and normal US	VTQ	Sperm concentration, and sperm motility	r _SWV_ = 0.614	r _SWV_ = 0.523
[40]	2018	60	31.55 ± 4.02	PE and CDUS	USE	Sperm concentration, and sperm motility	r _MS_ = −0.761	r _MS_ = −0.644
[42]	2017	50	30.00 ± 5.10	CDUS	USE	Sperm concentration	r _SR_ = 0.649	Undescribed
[43]	2017	48	-	PE and CDUS	SWE	Sperm count, total sperm motility, and sperm morphology	r _SI_ = −0.379	r _SI_ = −0.304
[39]	2016	92	30.00 ± 5.80	US and testicular tissue acoustic pulse radiation imaging	PE and SWE	Sperm concentration, and sperm motility	r _SWV_ = −0.728	r _SWV_ = −0.667
[41]	2016	60	26. 50 ± 5. 30	CDUS	USE	Sperm concentration, and sperm motility	r _MS_ = −0.773	Undescribed
[45]	2013	57	30.60 + 3.60	CDUS	Real-time tissue elastography	Sperm concentration, and sperm motility	r _MS_ = −0.773	r _MS_ = −0.638

*Abbreviations*: *PE* physical examination, *CDUS* color Doppler ultrasound, *USE* ultrasound elastography, *SWE* shear wave elastography, *VTQ* virtual touch quantification, *Emean* mean elastic modulus, *Emean-L* mean elastic modulus of the left testis, *Emean-R* mean elastic modulus of the right testis, *Emin* minimum elastic modulus, *Emin-L* minimum elastic modulus of the left testis, *Emin-R* minimum elastic modulus of the right testis, *Emax* maximum elastic modulus, *Emax-L* maximum elastic modulus of the left testis, *Emax-R* maximum elastic modulus of the right testis, *SWV* shear wave velocity, *MS* mean strain, *SI* stiffness index, *SR* strain ratio, *TH* testicular hardness, *LTSWE* left testicular shear wave elastic, *PSV* peak systolic velocity, *EDV* end-diastolic velocity, *RI* resistance index, *PI* pulse index, *E* elastic modulus

While USE parameters show promise for predicting semen quality in patients with VC, the limited number of studies and variability in instrumentation necessitate cautious interpretation. These elastography metrics should serve as adjunctive reference points rather than definitive predictors, guiding a more nuanced assessment of semen status in men with VC.

#### Color doppler ultrasound

CDUS is essential for diagnosing and monitoring VC, particularly through precise measurement of the intravascular diameter of the spermatic vein. We present the relevant data in Table 2. A larger diameter correlates with more severe reflux, longer reflux duration, and greater impairment of spermatogenesis. Position-dependent variations exist: the strongest association with abnormal semen parameters occurs when the maximal vein diameter exceeds 2.48 mm at rest and 2.63 mm during the Valsalva maneuver in the upright position [24]. One study found that the difference between the upright-rest and supine-rest diameters, using a 2.5 mm cutoff, was the best predictor of impaired spermatogenesis, significantly enhancing diagnostic accuracy. In contrast, another investigation reported no significant correlation between vein diameter and semen parameters in either position [46]. Hemodynamic CDUS metrics, such as maximum flow velocity and venous reflux time, generally show positive correlations with sperm count, concentration, and motility, independent of Valsalva maneuver. However, findings are inconsistent: Ediz et al*.*, in a small cohort of grade III VC patients, observed no significant correlation between Valsalva-measured vein diameter and semen parameters, likely due to limited sample size [47]. Mehraban reported that postoperative motility improvements correlated with preoperative vein diameters ≥ 2.5 mm and prolonged reflux times, whereas no significant changes were noted in veins smaller than 2.5 mm [48, 49].

**Table 2:** Color doppler ultrasound

Ref	Year	Patients	Mean age (year)	Diagnostic	Evaluation	Semen indicators	Correlation coefficient	
							Sperm concentration	Sperm motility
[50]	2023	72	29.57 ± 2.81	PE and CDUS	CDUS	Total sperm count, sperm concentration, and sperm motility	r _DR_ = 0.581 r _DV_ = 0.746 r _TR_ = 0.469 r _VS-Vmax_ = 0.405	r _DR_ = 0.325 r _DV_ = 0.302 r _TR_ = 0.290 r _VS-Vmax_ = 0.274
[47]	2021	84	Group 1: 27.50 Group 2: 24.50 Group 3: 26.00	PE and CDUS	CDUS	Semen volume, sperm concentration, progressive motility, immotile sperm, sperm morphology, sperm velocity, and pH	Undescribed	Undescribed
[51]	2019	60	31.77 ± 7. 48	PE and CDUS	CDUS	Sperm concentration, sperm motility, and sperm morphology	r _VD_ = −0.031	r _VD_ = 0.181
[46]	2018	30	31.03 ± 7.02	PE and CDUS	CDUS	Semen volume, appearance, sperm count, sperm motility, sperm morphology, and other microscopic details	r _ESV_ = 0.191 r _ISV A_ = −0.039 r _ISV B_ = 0.151	r _ESV_ = −0.073 r _ISV A_ = −0.289 r _ISV B_ = 0.759
[52]	2016	149	27.00 ± 5.70	PE and CDUS	CDUS	Semen volume, sperm concentration, sperm motility, and sperm morphology	Undescribed	Undescribed
[53]	2016	102	Group 1: 32.00 Group 2: 33.00 Group 3: 34.00	PE and CDUS	CDUS	Sperm concentration, and sperm motility	Undescribed	Undescribed
[54]	2015	60	30.01 ± 5.82	CDUS	CDUS	Sperm concentration, and sperm motility	r _SWV_ = −0.728	r _SWV_ = −0.667
[55]	2014	50	29.08 ± 5.42	CDUS	CDUS	Semen volume, sperm count, total sperm motility, progressive motility, and normal morphology	r _LTV_ = 0.579 r _RTV_ = 0.507	r _LTV_ = 0.287 r _RTV_ = 0.172
[24]	2014	87	27.00 ± 6.00	Real-time scrotal US	Real-time scrotal US	Semen volume, total sperm count, sperm concentration, total and progressive motility, and sperm morphology	Undescribed	Undescribed
[48]	2012	85	32.90	CDUS	CDUS	Sperm count, sperm motility, and sperm morphology	r _TVD_ = 0.200 r _RD_ = 0.370	r _TVD_ = 0.310 r _RD_ = 0.230
[56]	2011	80	31.20 ± 5.70	PE and CDUS	CDUS	Sperm concentration, and sperm motility	Undescribed	Undescribed
[57]	2010	93	30.90 ± 5.60	PE and CDUS	Power Doppler US	Sperm concentration, and sperm motility	r _PSV_ = −0.665 r _EDV_ = 0.143 r _Vm_ = 0.157 r _RI_ = −0.627 r _PI_ = −0.083	r _PSV_ = −0.382 r _EDV_ = −0.066 r _Vm_ = −0.001 r _RI_ = 0.236 r _PI_ = 0.081
[49]	2006	68	36.50	PE and High-resolution CDUS	High-resolution CDUS	Sperm count, sperm motility, and sperm morphology	Undescribed	Undescribed
[58]	1994	34	28.40	PE and CDUS	CDUS	Semen volume, sperm concentration, total sperm motility, progressive motility, sperm morphology, semen viscosity, and semen leukocyte content	Undescribed	Undescribed
[59]	1994	40	-	PE and CDUS	CDUS	Sperm concentration, sperm motility, and sperm morphology	Undescribed	Undescribed
[22]	1991	14	-	PE, CDUS and venography	CDUS and venography	Sperm concentration	Undescribed	Undescribed

*Abbreviations*: *PE* physical examination, *CDUS* color Doppler ultrasound, *DR* inside diameter, *DV* inner diameter at valsalva test, *VS-Vmax* maximum velocity, *TR* reflux time, *LTSWE* left testicular shear wave elastic, *PSV* peak systolic velocity, *EDV* end-diastolic velocity, *RI* resistance index, *PI* pulse index, *VD* vein diameter, *ESV* External spermatic vein, *ISV A* Internal spermatic vein A, *ISV B* Internal spermatic vein B, *SWV* shear wave velocity, *TVD* testicular vein diameter, *RD* reflux index, *Vm* mean velocity, *LTV* left testis volume, *RTV* right testis volume, *Vm* mean velocity

In men with VC-associated decreased semen quality, researchers have examined whether testicular arterial indices: resistance index (RI), pulsatility index (PI), and end-diastolic velocity (EDV) correlated with semen parameters in addition to standard spermatic vein measurements. Semiz et al*.* found no relationship between RI, PI, or EDV and semen parameters but did report a significant correlation between peak systolic velocity (PSV) and sperm count [55]. Interestingly, a negative PSV–sperm concentration correlation in the left subcapsular artery was also observed among control groups, perhaps reflecting thermoregulatory increases in testicular blood flow at slightly elevated temperatures. Following microscopic spermatic vein ligation in VC patients, RI and PI of the left testicular subcapsular artery were inversely correlated with sperm concentration and progressive motility, while EDV correlated positively with sperm concentration. In non-operated men, EDV maintained positive correlations with both concentration and motility, whereas RI and PI remained inversely related to these parameters. Han et al. corroborated these findings, demonstrating that PSV and RI of the left subcapsular artery can serve as predictive indices for reproductive outcomes [56]. Another study of men with azoospermia, oligozoospermia, or asthenozoospermia, reported elevated PSV and RI, further supporting their routine evaluation in infertile patients [60].

We propose that a change of 2.5 mm in spermatic vein diameter serves as the most reliable US predictor of impaired spermatogenesis in patients with VC. When this threshold is met, it indicates potential deterioration in semen quality, helping to identify individuals at heightened risk for fertility issues and guiding clinical decision-making. However, these correlations primarily reflect the hemodynamic impact of VC on testicular perfusion rather than acting as independent predictors of sperm parameters. Consequently, arterial indices (e.g., RI, PI, and EDV) are best utilized to confirm VC diagnosis or monitor treatment response, rather than as standalone predictors of semen quality. Although multiple studies have demonstrated that CDUS metrics have some utility in evaluating semen parameters in VC, variability in sample size and US settings precludes their use as definitive predictive tools. Larger, standardized studies are needed to validate the clinical applicability of these indices and improve their accuracy in forecasting semen quality among men with VC.

#### Contrast-enhanced ultrasound

Contrast-enhanced ultrasound (CEUS) is an emerging modality for VC evaluation, although studies remain limited. CEUS offers enhanced spatial resolution and superior hemodynamic assessment compared to conventional CDUS, particularly in mapping venous reflux patterns and microvascular perfusion [61]. Recent investigations have begun examining CEUS’s relationship to spermatogenic function in patients with VC [62]. For example, Zou et al*.* reported a significant negative correlation between venous wash-out time at 50% and key sperm parameters, including progressive motility and concentration. In contrast, other CEUS-derived metrics, such as arterial time in the spermatic artery, transit time from spermatic artery to vein, venous arrival time, venous peak time, and venous half-time, did not correlate significantly with semen parameters [63]. Quantitative CEUS parameters analyses thus show selective associations with semen quality indicators (Table 3).

**Table 3:** Contrast-enhanced ultrasound

Ref	Year	Patients	Mean age (year)	Diagnostic	Evaluation	Semen indicators	Correlation coefficient	
							Sperm concentration	Sperm motility
[63]	2023	40	31.00	PE and CDUS	CEUS	Sperm concentration, progressive motility, and total motility	r _TT_ = 0.027 r _ATA_ = −0.172 r _ATV_ = −0.159 r _PTV_ = 0.108 r _HTV_ = −0.163 r _WOT 50%_ = −0.755	r _TT_ = 0.026 r _ATA_ = −0.137 r _ATV_ = −0.125 r _PTV_ = 0.015 r _HTV_ = −0.225 r _WOT 50%_ = −0.683

*Abbreviations*: *PE* physical examination, *CDUS* color Doppler ultrasound, *CEUS* contrast- enhanced ultrasound, *ATA* arrival time in spermatic artery, *TT* transiting time from spermatic artery to spermatic vein, *ATV* arrival time in vein, *PTV* peak time in vein, *WOT 50%* wash-out time 50% in vein, *HTV* half time in vein

Although CEUS offers superior visualization of blood flow dynamics and microvascular alterations, CDUS remains the standard modality for routine VC assessment. The optimal clinical strategy is to employ CEUS as an adjunct to CDUS when detailed hemodynamic evaluation is necessary for comprehensive fertility assessment.

#### Diffusion-weighted imaging

Diffusion-weighted imaging (DWI) is an emerging investigational modality for VC diagnosis and management, offering enhanced assessment of testicular and epididymal changes associated with impaired venous reflux [64]. By quantifying the diffusion of water molecules within tissue, DWI can detect microstructural alterations, such as reduced perfusion, edema, or microvascular compromise that may be missed on conventional US [65]. Studies have shown significant correlations between lower apparent diffusion coefficient (ADC) values and impaired semen parameters (Table 4), indicating that VC-affected testicular tissue often exhibits decreased ADC values [66, 67].

**Table 4:** Diffusion-weighted imaging

Ref	Year	Patients	Mean age (year)	Diagnostic	Evaluation	Semen indicators	Correlation coefficient	
							Sperm concentration	Sperm motility
[66]	2019	30	28.90 ± 7.00	PE, CDUS, and MRI	DWI	Sperm count, sperm motility, and sperm morphology	r _MADC_ = 0.48	r _MADC_ = 0.33
[67]	2018	31	29.58 ± 8.12	Standard scrotal B-mode and CDUS	DWI	Semen volume, sperm concentration, sperm motility, and sperm morphology	r _ADC_ = 0.838	r _ADC_ = 0.417

*Abbreviations*: *PE* physical examination, *CDUS* color Doppler ultrasound, *MRI* magnetic resonance imaging, *DWI* diffusion-weighted imaging, *MADC* mean apparent diffusion coefficient, *ADC *apparent diffusion coefficient

Although DWI shows promise for VC diagnosis and semen quality assessment, existing studies have largely treated it as an adjunctive tool, yielding inconsistent results. Future research should involve multicenter clinical trials with larger cohorts to validate the relationship between DWI metrics and semen parameters and to clarify its role in monitoring VC treatment outcomes.

#### Laboratory tests

Laboratory evaluation of VC involves comprehensive assessment of biochemical and hormonal markers in relation to semen quality. Biomarkers examined include oxidative stress (OS) indicators (e.g., thiol–disulfide homeostasis), reproductive hormones (testosterone [T], follicle-stimulating hormone [FSH], luteinizing hormone [LH]), sperm acrosin activity, immunological factors (anti-sperm antibodies [AsAb]), and additional biochemical parameters such as prolidase, mean platelet volume (MPV), nitric oxide (NO), endothelin (ET), inhibin B (InhB), estradiol, prolactin, and seminal plasma proteins. Table 5 summarizes these markers’ correlations with semen parameters and evaluates their potential predictive value.

**Table 5:** Laboratory tests

Ref	Year	Patients	Mean age (year)	Diagnostic	Evaluation	Semen indicators	Correlation coefficient	
							Sperm concentration	Sperm motility
[68]	2024	246	Group 1: 27.80 ± 4.55 Group 2: 22.00 ± 1.81	PE and CDUS	MPV	Semen volume, progressive motility, sperm concentration, and sperm morphology	r _MPV_ = −0.262	r _MPV_ = −0.612
[50]	2022	72	29.57 ± 2.81	PE and CDUS	InhB, T, FSH, LH	Total sperm count, sperm concentration, and sperm motility	r _T_ = 0.675 r _LH_ = 0.435 r _FSH_ = 0.514 r _INHB_ = 0.793	r _T_ = 0.224 r _LH_ = 0.265 r _FSH_ = 0.337 r _INHB_ = 0.319
[69]	2022	80	30.12 ± 6.42	PE and CDUS	Serum NO, ET	Semen volume, sperm concentration, and progressive motility	r _NO_ = −0.634 r _ET_ = −0.251	r _NO_ = −0.404 r _ET_ = −0.284
[70]	2021	82	36.43 ± 8.53	PE and CDUS	Serum InhB	Sperm concentration, and sperm motility	r _INHB_ = 0.413	r _INHB_ = 0.362
[71]	2020	56	28.30 ± 5.50	PE and CDUS	TDH	Semen concentration, semen motility, and semen morphology	r _TT_ = −0.162 r _NT_ = −0.161 r _D_ = −0.107	r _TT_ = 0.334 r _NT_ = 0.297 r _D_ = 0.272
[72]	2020	112	Group 1: 32.50 ± 3.18 Group 2: 32.49 ± 3.15	PE and CDUS	Sperm acrosome enzyme activity	Sperm acrosomal enzyme activity, semen volume, sperm concentration, sperm motility, PR, and sperm morphology	r _AEA_ = 0.591	r _AEA_ = 0.632
[73]	2020	90	28.10 ± 3.20	CDUS	InhB, T, FSH and LH	Sperm concentration, sperm motility, and progressive motility	r _InhB VCI_ = 0.10 r _InhB VCII_ = 0.41 r _InhB VCIII_ = 0.45	r _InhB VCI_ = −0.06 r _InhB VCII_ = 0.34 r _InhB VCIII_ = 0.40
[74]	2016	54	Group 1: 16.30 ± 1.18 Group 2: 16.50 ± 1.12	PE	CRISP-3	Sperm concentration, total count, progressive motility, non-progressive motility, and morphology	Undescribed	Undescribed
[75]	2019	25	34.00 ± 9.00	PE	TPP1	Sperm concentration, total count, progressive motility, non-progressive motility, and morphology	Undescribed	Undescribed
[76]	2016	43	Group 1: 16.00 ± 2.00 Group 2: 16.00 ± 1.00	PE	IGFBP7	Semen volume, sperm concentration, total count, total motility, progressive motility, and morphology	Undescribed	Undescribed
[77]	2016	54	Group 1: 16.30 ± 1.18 Group 2: 16.50 ± 1.12	PE	CRISP-3	Semen volume, sperm concentration, total count, progressive motility, non-progressive motility, and morphology	Undescribed	Undescribed
[78]	2013	48	23.20	PE and CDUS	Prolidase enzyme activity	Semen volume, sperm concentration, sperm motility, and sperm morphology	r _PEA_ = −0.472	Undescribed
[79]	2006	20	32.00 ± 2.20	PE and CDUS	Hormone profile and AsAb	Semen volume, pH, sperm concentration, sperm motility, and sperm morphology	r _CC_ = 0.09	r _CC_ = 0.37
[80]	1989	11	28.90	PE and CDUS	T, FSH and LH	Sperm motility	Undescribed	Undescribed
[19]	1989	84	36.20	PE	Sperm-bound immunoglobulins and AsAb	Sperm concentration, sperm motility, sperm concentration, and sperm morphology	Undescribed	Undescribed

*Abbreviations*: *PE* physical examination, *CDUS* color Doppler ultrasound, *InhB* Inhibin B, *T* testosterone, *FSH* follicle-stimulating hormone, *LH* luteinising hormone, *E* estradiol, *PRL* prolactin, *MPV* mean platelet volume, *NO* nitric oxide, *ET* endothelin, *TT* Total thiol, *NT* native thiol, *D* disulphide, *AEA* acrosome enzyme activity, *PEA* prolidase enzyme activities, *CC* chromatin condensation, *AsAb* anti-sperm antibodies, *IGFBP7* insulin-like growth factor-binding protein 7, *CRISP-3* cysteine-rich secretory protein 3, *TPP1* tripeptidyl peptidase-1

#### Reproductive hormone

The seminiferous tubules’ functional architecture comprises Sertoli cells, which produce InhB, and spermatogenic cells, alongside interstitial Leydig cells that synthesize T. These elements work synergistically under the regulation of FSH and LH to sustain optimal spermatogenesis. However, VC’s precise effect on testicular endocrine function remains debated [81]. Studies exploring reproductive hormones in VC-associated decreased semen quality have produced inconsistent findings. Some report no significant differences in serum or seminal plasma InhB between VC patients and controls, whereas others demonstrate positive correlations between InhB levels, the InhB/FSH ratio, and key sperm parameters, such as concentration and progressive motility [82]. Conversely, FSH levels often correlate negatively with these semen parameters, suggesting FSH may be a more reliable marker for semen quality changes. Clinically, patients with InhB below 119.43 pg/mL, FSH above 3.86 mIU/mL, or an InhB/FSH ratio below 25.80 exhibit significantly higher rates of sperm abnormalities [50]. Although some investigations find no links between T, LH, or TSH ratios and semen parameters, other research indicates an inverse relationship between LH levels and spermatogenesis in VC patients [83]. These data imply distinct regulatory roles for LH and other reproductive hormones in VC-associated decreased semen quality. Therefore, assessing VC’s impact on fertility should encompass both semen analysis and hormonal profiling, particularly of FSH and InhB to more accurately evaluate testicular function and guide treatment decisions.

#### Anti-sperm antibodies

AsAb reflect an immunological response against sperm antigens, with higher prevalence observed in VC patients [84]. Elevated venous pressure and increased testicular temperature in VC can disrupt epididymal function and compromise the blood–testis barrier, facilitating immune complex deposition in testicular tissue [85]. Barrier breakdown exposes sperm antigens to the systemic circulation, inducing AsAb production. These antibodies may impair fertility via sperm agglutination, reduced motility, interference with spermatogenesis, and decreased penetration of cervical mucus [86].

#### Sperm acrosin

Sperm acrosin comprises trypsin-like serine proteases localized within the inner acrosomal membrane and plays an essential role in fertilization [87]. Upon gamete interaction, acrosin is activated and released to facilitate sperm-zona pellucida binding and penetration. Reduced acrosomal enzyme activity significantly impairs zona pellucida penetration, potentially leading to infertility [88]. Observational studies have shown positive correlations between acrosin activity and semen parameters such as sperm concentration, progressive motility, and normal morphology. VC has been shown to decrease acrosin activity, thereby adversely affecting overall semen quality [72]. Consequently, assessing acrosin activity provides valuable insights into sperm functional capacity, informs treatment monitoring, and aids in prognostication for VC patients.

#### Mean platelet volume

Recently, platelet-related markers have been extensively investigated in various vascular disorders. Elevated MPV has been associated with VC, potentially reflecting endothelial dysfunction in affected individuals [89, 90]. However, findings are inconsistent, with some studies reporting no significant differences in platelet indices between VC patients and controls [91]. MPV is also implicated in inflammatory processes and prognostic assessments, both of which can adversely impact semen quality [92]. The increased MPV observed in VC-associated decreased semen quality may result from inflammation and OS driven by platelet activation [93]. Correlation analyses indicate that higher MPV negatively affects sperm concentration, motility, and morphology by promoting platelet aggregation and endothelial dysfunction. Notably, MPV exhibits considerable diagnostic sensitivity for predicting semen quality in men with VC [68].

Although MPV shows potential clinical relevance, it remains an emerging biomarker in VC research and requires further validation as an independent predictor. Post-treatment monitoring of MPV via routine hematological assessment may offer additional insights into inflammatory status and corresponding changes in semen quality. As an easily obtainable marker, MPV could complement established diagnostic modalities, such as US and semen analysis, potentially enabling earlier detection of VC-associated decreased semen quality and more timely evaluation of treatment response.

#### Serum nitric oxide and endothelin

NO, primarily synthesized by endothelial NO synthase, is a key vasodilator. Under physiological conditions in testicular tissue, NO supports sperm motility and metabolic regulation. However, pathological elevations in NO can impair vasodilatory balance, disrupt testicular and epididymal perfusion, and compromise spermatogenesis, leading to reduced sperm function and potential infertility [94]. ET also contributes to spermatogenesis and sperm maturation. Elevated ET levels promote localized vasoconstriction, worsening venous stasis and further reducing testicular blood flow, with adverse effects on sperm quality. Studies have demonstrated negative correlations between serum NO and ET levels and sperm concentration and progressive motility [69]. Taken together with VC pathophysiology, NO and ET may serve as valuable biomarkers for gauging VC severity and its impact on spermatogenic function.

#### Thiol–disulphide homeostasis

Research on the relationship between VC and TDH is limited. OS is a key pathogenic mechanism in VC-associated decreased semen quality, and TDH serves as an important marker of oxidative status. Measurements of native thiol, total thiol, and disulfide levels taken preoperatively and six months after VC surgery, show positive correlations with both progressive and non-progressive motility [71]. Although these correlations are modest, they underscore OS’s role in VC-associated infertility. Postoperative reductions in OS and corresponding TDH normalization suggest that TDH may be a useful indicator for assessing oxidative status and evaluating surgical outcomes.

#### Prolidase enzyme

Prolidase, an enzyme integral to proline recycling during collagen synthesis and cellular growth, has been studied in various pathological contexts, but data on its role in VC are sparse [95–97]. Preliminary findings suggest a negative correlation between prolidase enzyme activity in the varicose vein wall and sperm count in men with VC-associated infertility, implying a potential contribution to infertility progression [78, 98]. However, limitations such as the lack of control group comparisons and stratification by VC grade, preclude the use of prolidase activity as a definitive predictor of semen quality changes in this population.

#### Seminal plasma proteins

Seminal plasma proteins have emerged as potential biomarkers of impaired spermatogenesis and could indicate the need for varicocelectomy before VC is apparent on semen analysis [99]. Zylbersztejn et al*.* applied proteomics profiling to adolescent VC patients and identified 30 differentially expressed proteins. They concluded that proteomics could characterize seminal plasma proteins, facilitating the discovery of novel markers of spermatogenesis and sperm function [100]. By reflecting the presence of VC through specific protein signatures, the seminal plasma proteome may enhance the sensitivity of early fertility impairment detection and guide timely therapeutic intervention.

Cysteine-rich secretory protein 3 (CRISP-3), a member of the CRISP family involved in inflammatory processes, is elevated in the seminal plasma of men with VC and decreases following varicocelectomy. In human seminal plasma, CRISP-3 binds β-microsemino protein and is secreted throughout the male reproductive tract [77]. Proteomic analyses in adolescents have shown that VC can increase seminal CRISP-3 levels by up to 90-fold in association with declining semen quality [74]. Subsequent studies in adults confirmed a pronounced elevation of primarily the unglycosylated form of CRISP-3 in VC patients, with levels falling and semen parameters improving after surgical correction [101]. Monitoring CRISP-3 levels may therefore aid in evaluating fertility changes in men with VC.

In adolescents, seminal plasma levels of insulin-like growth factor-binding protein 7 (IGFBP7), a regulator of cell growth, differentiation, and proliferation that also stimulates prostacyclin (PGI₂) production are elevated in grade II and III varicoceles [76]. Aberrant increases in PGI₂, a potent vasodilator, may exacerbate hemodynamic disturbances and oxidative damage in VC patients. Additionally, tripeptidyl peptidase-1 (TPP1) has emerged as a potential predictor of surgical outcome: adults with favorable responses to varicocelectomy exhibit approximately a threefold rise in TPP1 levels post-operatively1 [75]. Although TPP1’s serine protease activity is implicated in conditions such as ischemia and inflammation, its role in fertility remains unclear.

We propose simultaneous assessment of seminal CRISP-3, IGFBP7, and TPP1 alongside routine clinical tests to pinpoint the optimal timing for intervention and enhance fertility prognoses in men with VC.

#### A general proposed guideline for varicocele

To address practice heterogeneity and standardize clinical stratification, we propose the following stepwise guideline for VC diagnosis:

1. Obtain a focused history from all men presenting with infertility or scrotal discomfort, assessing risk factors such as family history, previous inguinal surgery, and occupational exposures.
2. Perform a standardized PE in both supine and standing positions, including visual inspection, palpation, and the Valsalva maneuver. Grade findings I–III based on palpability and visibility at rest or during strain [12].
3. If PE reveals a Grade II–III VC, proceed directly to US for quantitative evaluation [16].
4. If clinical suspicion persists despite a negative or equivocal PE, especially in resource-limited settings without expert palpation, perform US to avoid underdiagnosis [17].
5. On US, a maximal spermatic vein diameter > 3 mm in the upright position or a reflux duration > 2 s during the Valsalva maneuver indicates clinically significant VC [24].
6. When US findings are borderline or additional functional data are needed, USE provides objective tissue-stiffness measurements. A testicular elastic modulus exceeding 5.235 kPa correlates with impaired spermatogenesis and can guide management of subclinical cases [34].
7. In specialized centers, CEUS and DWI can be reserved for research protocols or to resolve equivocal findings by characterizing microvascular perfusion and tissue diffusion properties, respectively [62, 63].

Finally, we recommend integrating these imaging criteria into a composite report that encompasses PE grade, vein diameter and reflux metrics, and elastography values. While this algorithm offers a structured and evidence-informed framework for the assessment of VC-associated decreased semen quality, it is important to emphasize that PE remains the cornerstone of VC diagnosis, as acknowledged throughout this review. Imaging and laboratory modalities provide valuable complementary information, but their precise clinical utility and predictive value are not yet fully established. Therefore, this proposed diagnostic approach should be regarded as a preliminary framework intended to guide further research rather than a definitive standard. Its implementation and refinement will benefit from prospective, longitudinal studies across diverse clinical settings to enhance its evidence base and clarify its prognostic and therapeutic implications.

### Limitations

1. Methodological Heterogeneity: Included studies exhibited substantial variation in measurement techniques, diagnostic criteria, and reporting standards, particularly for US parameters, making direct comparisons.
2. Sample Size Variations: Many studies, especially those on novel biomarkers, had relatively small sample sizes, limiting the generalizability of their findings.
3. Geographic Bias: Although our review encompassed both English and Chinese databases, it may not fully capture research from other regions or non-English/Chinese publications.
4. Temporal Limitations: The search extended through January 2025, rapid advances in imaging techniques may mean newer methodologies were not included.
5. Limited Long-term Follow-up: Most studies were cross-sectional, preventing thorough evaluation of the prognostic value of diagnostic tools over time.
6. Standardization Issues: The lack of universally accepted diagnostic criteria for VC, particularly in US assessment, complicated evidence synthesis across studies.

## Conclusions

This review offers comprehensive insights into diagnostic strategies for assessing the relationship between VC and semen parameters. Imaging techniques, particularly USE and CDUS, show significant promise in evaluating VC's impact on semen quality. Key threshold values, such as testicular elastic modulus exceeding 5.235 kPa and a spermatic vein diameter change of 2.5 mm, serve as clinically relevant markers for predicting semen impairment. Laboratory tests, although exhibiting variable correlations with semen parameters, provide valuable complementary data for a holistic patient assessment. Emerging biomarkers, such as CRISP-3 and TPP1, merit validation in large studies. Based on current evidence, we recommend prioritizing imaging techniques for predicting semen quality abnormalities. Early detection of sperm impairment can be achieved by dynamically monitoring key imaging indicators, such as blood flow parameters and elastography values. In addition, combined laboratory tests including reproductive hormone, seminal plasma proteins should serve as adjunctive diagnostic tools. Integrating these modalities allows the development of a multimodal stratification. Future research should aim to establish standardized diagnostic protocols and develop integrated assessment frameworks that combine multiple modalities, thereby enhancing the precision of VC evaluation and informing better clinical decision-making for men with VC-associated decreased semen quality.

## Data Availability

No datasets were generated or analysed during the current study.

## Abbreviations

ADC: Apparent diffusion coefficient
ARFI: Acoustic radiation force impulse
AsAb: Anti-sperm antibodies
ASRM: American Society for Reproduction Medicine
AUA: American Urological Association
CDUS: Color Doppler ultrasound
CEUS: Contrast-enhanced ultrasound
CRISP-3: Cysteine-rich secretory protein 3
DWI: Diffusion-weighted imaging
EAU: European Association of Urology
EDV: End-diastolic velocity
ET: Endothelin
FSH: Follicle-stimulating hormone
IGFBP7: Insulin-like growth factor-binding protein 7
InhB: Inhibin B
LH: Luteinizing hormone
MPV: Mean platelet volume
MRI: Magnetic resonance imaging
NO: Nitric oxide
OS: Oxidative stress
PE: Physical examination
PGI2: Prostacyclin
PI: Pulsatility index
PSV: Peak systolic velocity
RI: Resistance index
ROC: Receiver operating characteristic
SWE: Shear wave elastography
SWV: Shear wave velocity
T: Testosterone
TDH: Thiol-disulphide homeostasis
TPP1: Tripeptidyl peptidase-1
US: Ultrasound
USE: Ultrasound elastography
VC: Varicocele
